# Special Issue “Diagnosis and Treatment of Rare Diseases”

**DOI:** 10.3390/jcm13092574

**Published:** 2024-04-27

**Authors:** Álvaro Hermida-Ameijeiras

**Affiliations:** Department of Psychiatry, Radiology, Public Health, Nursing and Medicine, University of Santiago de Compostela, 15704 Santiago de Compostela, Spain; alvaro.hermida@usc.es

Rare diseases (RDs) represent a large and heterogeneous group of low-prevalence conditions, and 473 million people could be affected worldwide. A total of 71.9% of these conditions are genetic, and 69.9% have an exclusively paediatric onset, often causing a variable degree of disability and impact the patients’ and caregivers’ quality of life [1,2]. The early diagnosis of these diseases would allow the establishment of an adequate treatment, and thereby minimize or avoid serious complications with irreversible consequences. However, there is often a delay in diagnosis, and 25% of patients wait between 5 and 30 years for the disease to be diagnosed [3]. To increase awareness among healthcare professionals, characterize patients, and enhance clinical and molecular diagnosis for a greater number of people with undiagnosed RDs, the Journal of Clinical Medicine (JCM) dedicates this Special Issue focused on “Diagnosis and Treatment of Rare Diseases”.

To improve the knowledge of RDs, it is necessary to disseminate the information that is available. Long-term follow-up is the key priority. Some clinical characteristics, such as acanthosis nigricans, the generalised absence of adipose tissue (particularly from the palms and soles), together with phlebomegaly, may be used to differentiate acquired generalised lipodystrophy (AGL) from other lipodystrophy subtypes, according to the description of seven patients in a 14-year-long follow-up [4]. It is also remarkable that all these patients exhibited autoimmune conditions. A second study fully described the main neurological and dermatological features of Tuberous Sclerosis Complex (TSC) in 32 adult patients. As a result, a positive correlation between the presence of cutaneous angiofibromas and epilepsy was found. The authors also suggest the potential prognostic value of subependymal nodules for intellectual decline [5]. An attempt to better characterize rare disorders using a histologic approach was conducted on 11 patients with Fabry disease and the p.Gln279Arg variant in the GLA gene [6]. All the patients were diagnosed with the classical phenotype, supported by histological hallmarks. Repeated ultrastructural studies highlighted the benefit of prompt therapy.

Novel approaches to solving complex problems include molecular medicine and artificial intelligence, as several contributors remark. The first study pointed out the utility of saliva to screen for primary sclerosing cholangitis (PSC). Twenty-five proteins were differentially expressed among patients with PSC when compared to a healthy control group. The potential diagnostic value of disulfide-isomerase A3 and peroxiredoxin-5 has been suggested [7]. The second study attempted to obtain detailed characteristics about patients with Fabry disease, retrospectively evaluating 19,385 patients’ electronic health records (EHRs). It was described how natural language processing (NLP) may support physicians to ameliorate diagnosis delays and misdiagnosis by developing a decision-support scoring system [8]. It identified one patient at high risk of FD, and later confirmed the diagnosis after performing a DBS assay. Finally, one more study underlined the importance of using bioinformatics and genomics to better understand the link between genotypes and phenotypes and the functional impact of genetic variations. The reported case of a 59-year-old male patient with features typical of Tangier disease and homozygous for a novel variant in the ABCA1 gene (c.4799A > G; p. His1600Arg) clearly demonstrated the utility of in silico analysis to predict the pathogenicity of novel variants and how protein models generated using the SWISS-MODEL allow for the characterization of the changes in structure and functionality due to the reported variants [9].

Clearly, more knowledge of the mechanisms of action of drugs and their associated biological effects is required in the field of RDs. On 10 March 2023, Trofinetide obtained approval from the US FDA as the first Rett syndrome treatment. But its mechanism of action (improving neuronal morphology and synaptic functioning) may be better suited to treat other neurodevelopmental disorders (such as Fragile X syndrome or autism spectrum disorders). The implementation in the next years of Trofinetide-based molecules or innovative drug-formulations will contribute to personalize therapies and increase options for these neurological rare disorders [10]. One additional example of using an individual approach to overcome new challenges is the first-reported case of enteral nutrition for an adult patient with PKU severe dysphagia. The authors faced complications, including the enteral feeding tubes clogging and gastrointestinal issues (vomiting and diarrhoea due to hyperosmolality); therefore, they needed to combine PKU oral supplementation based on CGMP or Phe-free L-amino acid supplements together with standard enteral nutrition products. Thus, the caloric and protein intake requirements were fully met [11].

Finally, the physical, emotional, and economic impacts that patients with RDs and their caregivers suffer from are remarkable, and this was shown throughout a cross-sectional questionnaire addressed to 16 parents of children with Menkes disease (MD). According to the participants, comorbidities have a large impact on both the emotional functioning of patients with MD and the cognitive functioning of the patient’s family [12]. One of the main physical impact is the diagnosis delay. Patients with Obstructed Hemivagina and Ipsilateral Renal Agenesis/Anomaly (OHVIRA) Syndrome are also at high risk of acute complications if it is diagnosed lately. The authors of one case study concluded that the risk of possible complications can be effectively prevented by performing diagnostic pelvic imaging at the time of diagnosis of renal agenesis, or at the beginning of puberty [13].

Specialized centres for rare diseases must be unequivocally multidisciplinary, since this is the best way to share opinions and make decisions, as was reflected in the case report of a 28-year-old woman with Lysinuric protein intolerance (LPI) who had experienced three early miscarriages. The patient finally became pregnant, and despite diverse nutritional and medical challenges (food aversion, intrauterine growth restriction, bleeding risk, and preeclampsia suspicion), successfully delivered. This is conceivable thanks to the multidisciplinary approach [14].

As the Guest Editor, I would like to give special thanks to the reviewers for their thoughtful comments, which improved the manuscripts, and to the JCM editorial team for their support. Finally, I sincerely thank all the authors for their valuable contributions.
